# Development of Genome Editing Approaches against Herpes Simplex Virus Infections

**DOI:** 10.3390/v13020338

**Published:** 2021-02-22

**Authors:** Isadora Zhang, Zoe Hsiao, Fenyong Liu

**Affiliations:** 1 School of Public Health, University of California, Berkeley, CA 94720, USA; isadoraz@berkeley.edu (I.Z.); zhsiao@berkeley.edu (Z.H.); 2 Department of Molecular and Cell Biology, University of California, Berkeley, CA 94720, USA; 3 Program in Comparative Biochemistry, University of California, Berkeley, CA 94720, USA

**Keywords:** herpesvirus, herpes simplex virus, genome editing, homing endonuclease, CRISPR, Cas9

## Abstract

Herpes simplex virus 1 (HSV-1) is a herpesvirus that may cause cold sores or keratitis in healthy or immunocompetent individuals, but can lead to severe and potentially life-threatening complications in immune-immature individuals, such as neonates or immune-compromised patients. Like all other herpesviruses, HSV-1 can engage in lytic infection as well as establish latent infection. Current anti-HSV-1 therapies effectively block viral replication and infection. However, they have little effect on viral latency and cannot completely eliminate viral infection. These issues, along with the emergence of drug-resistant viral strains, pose a need to develop new compounds and novel strategies for the treatment of HSV-1 infection. Genome editing methods represent a promising approach against viral infection by modifying or destroying the genetic material of human viruses. These editing methods include homing endonucleases (HE) and the Clustered Regularly Interspaced Short Palindromic Repeats (CRISPR)/CRISPR associated protein (Cas) RNA-guided nuclease system. Recent studies have showed that both HE and CRISPR/Cas systems are effective in inhibiting HSV-1 infection in cultured cells in vitro and in mice in vivo. This review, which focuses on recently published progress, suggests that genome editing approaches could be used for eliminating HSV-1 latent and lytic infection and for treating HSV-1 associated diseases.

## 1. Introduction

Herpes simplex virus 1 (HSV-1), also known as human herpesvirus 1, is the prototype member of the human herpesvirus family, which also includes herpes simplex virus 2 (HSV-2), Varicella Zoster virus (VZV), Epstein–Barr virus (EBV), human cytomegalovirus (HCMV), human herpesviruses 6a and 6b, human herpesvirus 7, and Kaposi’s sarcoma-associated herpesvirus (KSHV) [1,2]. Like other herpesviruses, HSV-1 contains a double-stranded DNA genome which is packaged in an icosahedral shaped capsid. HSV-1 infectious particles, or virions, possess a lipid envelope that contains viral surface glycoproteins and encases the tegument compartment and the viral capsid [3].

Like all other herpesviruses, HSV-1 can engage in lytic infection to produce viral progeny as well as establish latent infection that can co-exist with the host during the lifetime of the host [1,2]. As a ubiquitous virus with seroprevalence of more than 50% of the US population, HSV-1 is the causative agent for cold sores and is one of the most common causes of infectious blindness (keratitis) and viral encephalitis [4,5]. This virus usually causes mild and sometimes asymptomatic infections in healthy or immunocompetent individuals but can lead to severe and potentially life-threatening complications in immune-immature individuals such as neonates or immune-compromised patients [2].

As in all other herpesviruses, the lytic infection of HSV-1 starts with the step of viral entry through interactions of the viral surface glycoproteins with the cellular receptors [1,2]. Following the viral entry step, viral DNA genome is located to the nuclei. Viral genes are expressed in a cascade fashion, defined by immediate-early (IE), early (E), and late (L) phases. Viral genomes are replicated, and capsids are assembled. Finally, viral infectious particles or virions are formed and released to the extracellular space [2]. The entire cycle of lytic infection is tightly regulated by a collection of viral genes (e.g., *ICP4* and *UL54*), which are essential for HSV-1 replication and lytic infection and represent antiviral targets for drug development [2]. After primary infection at the mucosal surfaces, HSV-1 establishes lifelong latency in sensory neurons, including those of the trigeminal ganglia (TG) and autonomic ganglia, from which they can later reactivate, causing recurrent lesions. The mechanisms of HSV-1 latency initiation and persistence are not fully understood. Certain HSV-1 gene products (e.g., viral latency-associated transcript (LAT) and other non-coding RNAs in the LAT locus) are either found to express or play a role in these processes [2].

Standard HSV-1 therapy consists of nucleotide analogs such as acyclovir, valacyclovir, and famciclovir [6,7]. These drugs function to inhibit viral DNA synthesis and are highly effective in blocking viral replication and lytic infection [2]. However, they have little effect on viral latency and cannot completely eliminate viral infection [6,7]. These issues, along with the emergence of drug-resistant viral strains, pose a need to develop new compounds and novel strategies for the prevention and treatment of HSV-1 infection. This review focuses on recent research progress in developing genome editing-based approaches against HSV-1 infections by targeting and eliminating HSV-1 genomes during both lytic and latent infections.

## 2. Genome Editing Technologies and Approaches

Genome editing methods represent a promising and novel approach against viral infection and replication by modifying or destroying the genetic material of human viruses [8,9,10,11]. These editing methods include homing endonucleases (HE; i.e., meganucleases), zinc finger nucleases (ZFN), transcription activator-like effector nucleases (TALEN), the Clustered Regularly Interspaced Short Palindromic Repeats (CRISPR)/CRISPR associated protein (Cas) RNA-guided nuclease system, and engineered tyrosine recombinases (e.g., Cre variants). Based on their mode of action, HE, ZFN, TALEN, and CRISPR/Cas systems (e.g., CRISPR/Cas9) engage in error-prone repair-mediated DNA modifications while error-free repair is employed in site-specific recombinase systems such as the Cre-based method [8,9,10,11].

**Figure 1:**
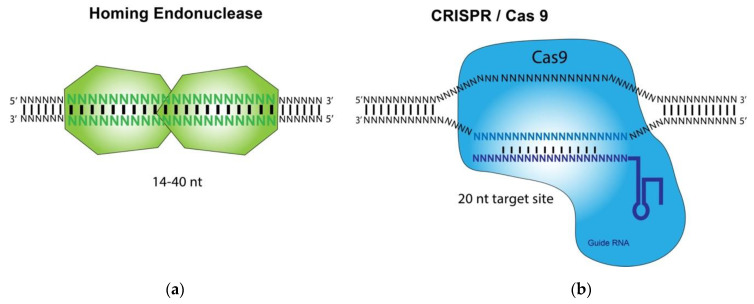
Illustration of the homing endonuclease (HE, meganuclease) and CRISPR/Cas9 System: (**a**) Homing endonuclease-mediated recognition and binding of a DNA substrate leads to the cleavage of the substrate.; (**b**) A custom-designed guide RNA (gRNA) hybridizes its target sequence and directs Cas9 endonuclease to cleave a DNA substrate.

HE, ZFN, TALEN, and CRISPR/Cas9 are called designer nucleases and differ primarily in the mode of how these nucleases recruit their DNA target sites (Figure 1). HE, ZFN, and TALEN recognize their target DNA sequences by using intrinsic DNA binding domains [8,9,10,11]. In contrast, the CRISPR-associated Cas9 nuclease is guided to a DNA cleavage site by interacting with a short guide RNA (gRNA) that hybridizes to its target DNA site of about 20 bp [12]. The base pairings between the target DNA and the gRNA, which is recognized by Cas9, dictate the binding specificity of the CRISPR/Cas9 technology. Studies using HE and CRISPR/Cas9 for gene targeting applications, such as anti-HSV-1 infection, have been carried out and reported [8,9,10,11]. In this review, we will focus on the recent developments in the use of these two methods for inhibition and treatment of HSV-1 infections.

Homing endonucleases represent a promising class of DNA-cleaving enzymes [13]. Unlike the ZFN and TALEN, HE is much smaller (~250 amino acids) because the regions responsible for DNA-cleaving activity and DNA binding are the same [10]. Another unique aspect of these enzymes is that they exhibit high sequence specificity for their DNA targets and therefore, in principle, will display fewer off-target effects [13].

A major challenge in the use of HE technology for genome editing is redirecting their cleavage specificity to the desired DNA targets [13]. Their DNA binding interaction and sequence specificity are strongly influenced by the neighboring amino acids and DNA contacts. Moreover, the fact that both DNA binding and cleavage activity reside within the same protein regions make engineering of these enzymes difficult because alterations to improve DNA binding and specificity could impair the cleavage activity of the HEs [10]. As a result, complex, tedious, and challenging modifications and engineering are needed in order to develop meganucleases with unique and novel binding specificities [13].

In contrast to HE, ZFN, and TALEN, the CRISPR-associated Cas9 nuclease is guided to a DNA cleavage site by interacting with a short guide RNA (gRNA) that hybridizes to its target DNA site of about 20 bp [10]. CRISPR/Cas9 RNA-guided nucleases are derived from an adaptive immune system that evolved in bacteria to defend against invading plasmids and viruses [9]. In a CRISPR system, short sequences of invading nucleic acids are incorporated into CRISPR loci. They are then transcribed and processed into CRISPR RNAs (crRNAs) which, together with a trans-activating crRNAs (tracrRNAs), complex with CRISPR-associated (Cas) proteins to dictate the specificity of DNA cleavage by Cas nucleases through base pairing interactions between these nucleic acids [12]. Doudna, Charpentier, and colleagues carried out in vitro DNA cleavage experiments and showed that this system can be reduced to two components by fusion of the crRNA and tracrRNA into a single guide RNA (gRNA) [9]. Furthermore, they showed that re-targeting of the Cas9/gRNA complex to new sites could be accomplished by altering the sequence of the gRNA [12].

Unlike HE, ZFN, and TALENs, CRISPR/Cas nucleases do not need to change the Cas protein in order to recognize each DNA target site. The gRNA sequence dictates the specificity of the CRISPR/Cas system [12]. Since the gRNA does not covalently link to the Cas9 protein, this system is highly amenable to multiplexing through the concurrent use of multiple gRNAs to induce double strand breaks (DSBs) at several loci. Additional studies on these issues have demonstrated the utility of the CRISPR/Cas9 technology for genome-editing applications [12].

## 3. Development of CRISPR/Cas9-Based Technologies against HSV-1 Infection

Roehm et al. reported the first study using the CRISPR/Cas9 system against HSV-1 productive infection in cell culture [14]. Three guide RNAs were constructed to target different regions of the viral DNA that encode HSV-1 ICP0 protein. ICP0 is an important HSV-1 immediate early (IE) regulatory protein with significant impact on viral gene expression and replication [2]. In an ICP0-complementing L7 cell line, the CRISPR/Cas9 system with the constructed gRNAs was effective in generating mutations at the target *ICP0* DNA sequences, including frameshift and deletion mutations, and reducing the expression of ICP0 protein.

To assess the impact of these mutations on the ability of *ICP0* to stimulate HSV-1 replication, experiments were carried out to compare *ΔICP0* HSV-1 replication levels in cell lines expressing the *ICP0* mutants to those seen in control cells expressing the wild type ICP0 protein [14]. These results indicated that cells expressing a functional gRNA and Cas9 showed marked declines in their ability to support the replication of *ΔICP0* HSV-1, indicating Cas9/gRNA-mediated inactivation of *ICP0* in these cells. Cas9/gRNA-induced mutations of *ICP0* were found to interfere with its PML body-disrupting antiviral activity [14].

To investigate the anti-HSV-1 activity of the CRISPR/Cas9 system in human cells, clonal cell lines expressing Cas9 and gRNAs were derived from human oligodendroglioma cell line TC620 and were infected with HSV-1 [14]. Biochemical and fluorescence microscopy studies indicated inhibition of ICP0 protein production and suppression of HSV-1 infection and replication. Plaque assays showed that viral titers and growth were reduced in cells expressing Cas9 and gRNAs.

Expression of Cas9 and gRNAs had no significant effect on cell cycle progression, apoptosis, or cell viability of TC620 cells. SURVEYOR assays and PCR sequencing analyses showed little off-target effects of the anti-HSV-1 Cas9/gRNA systems as no indel mutations were found in several representative human genes that were identified by bioinformatics screening analyses. These results suggest that the Cas9/gRNA system exhibits little cytotoxicity or off-target effects [14]. Additional experiments using lentivirus-mediated delivery of gRNAs simultaneously targeting HSV-1 essential genes *ICP4* and *ICP27* in addition to *ICP0* showed that HSV-1 infection was almost completely blocked in these cells. These results demonstrate the potential of using the Cas9/gRNA system for inhibiting HSV-1 lytic and productive infection in vitro [14].

In a series of elegantly designed experiments, van Diemen and colleagues presented interesting results showing that CRISPR/Cas9-mediated genome editing methods can limit the infection and replication of three human herpesviruses, HSV-1, human cytomegalovirus (HCMV), and Epstein–Barr virus (EBV) [15]. In the experiments with HSV-1, gRNAs were constructed to target 12 HSV-1 essential genes and two nonessential genes.

Vero cells were introduced with anti-HSV-1 gRNAs and then infected with HSV-1-eGFP that contained a GFP expression cassette, and GFP expression was monitored as a measure for HSV-1 infection and replication [15]. Most gRNAs targeting essential HSV-1 genes impaired viral replication efficiently. Interestingly, targeting of non-essential genes (i.e., *US3* and *US8*) also reduced HSV-1 replication, albeit to a lesser extent as compared to targeting essential genes. This can be partially explained as the Cas9/gRNA system generated double strand DNA breaks, leading to inactivation of these genomes for viral progeny production, even though the nonessential genes are targeted [15].

Treatment with single gRNAs targeting essential genes appeared to be less effective in reducing HSV-1 growth than treatment with double gRNAs targeting combinations of these genes. For example, a single gRNA targeting *UL52* resulted in a 10,000-fold reduction in virus titer. Combining two gRNAs targeting *UL29* and *UL8* or *UL29* and *UL52* caused an almost complete loss of infectious viral particles, with a 1,000,000-fold reduction in virus titer [15].

To assess potential off-target editing by the CRISPR/Cas9 system, the activity of 9 gRNAs in modifying the top three predicted off-target sites within the human genome was analyzed. These 27 loci of the human genome in DNA samples isolated from gRNA-expressing and control cells were amplified by PCR and subjected to sequencing analysis. No signs of CRISPR/Cas9-induced editing were detected at these loci, suggesting that the CRISPR/Cas9-mediated genome editing did not occur at undesired sites [15].

The activity of these constructed anti-HSV-1 gRNAs was showed to abrogate the replication of HSV-1 reactivated from quiescence, using a unique and interesting cultured cell model for viral quiescent infection [15]. HSV-1 replication upon reactivation was inhibited in the cultured cell model expressing functional gRNAs. These results are consistent with another recent study using the CRISPR/Cas9 systems against HSV-1 lytic infection in Vero cells [16].

In a recent study, David Knipe and colleagues examined the effect of the CRISPR/Cas9 system against HSV-1 infection and investigated the mechanism of how Cas9/gRNA cleaved the target lytic and latent viral genomes in cultured cells [17]. It had previously been shown that CRISPR/Cas9 editing required efficient access to target sites within chromatin [18,19,20]. Previous studies indicated that differential host cell mechanisms regulating chromatin assembly on viral DNA in permissive (epithelial cells) versus non-permissive (neuronal) cells contribute to the lytic versus latent infection decision by HSV-1 [21]. HSV-1 DNA during latency has a more compacted chromatin structure than replicating viral DNA, leading to less Cas9/gRNA access to DNA target sites [18,19,20].

To address these issues, a collection of gRNAs was constructed to target 4 viral essential genes (i.e., *UL29*, *UL30*, *UL54*, and *ICP4*)[17]. These gRNAs were screened in an in vitro SaCas9-based cleavage assay to identify gRNAs that could efficiently target lytic and quiescent HSV-1 genomes in human cells. SaCas9 was derived from *Staphylococcus aureus* and is small enough to be encoded by AAV-based vector delivery systems [22]. In human foreskin fibroblasts infected with HSV-1, many constructed gRNAs were effective in reducing HSV-1 replication [17], confirming previous observations [14,15] that Cas9/gRNA system can inhibit HSV-1 lytic infection.

To study the effect of the constructed gRNAs on viral latent genome, Knipe and colleagues used a HSV-1 quiescent infection system with replication-defective HSV-1 d109 virus, which showed heterochromatin loading in viral DNA [23] similar to murine latent infection [24,25]. Experiments with this quiescence infection model indicated that anti-HSV-1 gRNAs could reduce reactivation of quiescent d109 genomes, with combinations of two different gRNAs exhibiting an additive effect [17]. Furthermore, these results showed that CRISPR/Cas9 induced a substantial level of indel mutations in quiescent HSV-1 genomes. Thus, gRNAs that target quiescent HSV-1 genomes efficiently were identified and SaCas9/gRNA-mediated indel mutations led to loss-of-function mutations in the corresponding proteins [17]. Further experiments showed CRISPR/Cas9 can edit both input (non-replicating) and replicating genomes, although it edits the replicating genomes much more efficiently.

Analyses of the mutations and target regions indicated that cleaved quiescent d109 genomes were re-joined without induction of significant loss of sequences or viral DNA degradation during the quiescent infection. However, sequencing analysis indicated that editing of lytic HSV-1 genomes caused extensive loss of viral sequences around the target sites. Thus, the edited viral genomes during quiescent and lytic infections exhibited different patterns of mutations.

To investigate if Cas9/gRNA targeting affects HSV-1 infection in vivo, Jerome and colleagues used adenovirus associated virus (AAV)-based vectors to deliver Cas9 and gRNA expression cassettes in a mouse model of HSV-1 ocular infection [26]. SaCas9 and gRNAs targeting *UL30* and *UL54* were used. Several gRNAs were able to promote substantial levels of gene editing on HSV-1 genomes in latently-infected cultured neurons transduced by SaCas9/gRNA-expressing AAV vectors [26].

In mouse experiments, latent HSV-1 infection was established in mice through ocular infection, followed by delivery of AAV vectors carrying SaCas9 and gRNA expressions cassettes. Trigeminal ganglia (TG) neurons were isolated and analyzed [26]. Consistent with their results with single HE treatment, PCR quantification showed similar HSV-1 levels in the neurons of treated and control animals [26,27]. In contrast to the easily detected gene editing of HSV-1 after single-HE treatment, Cas9/gRNA-mediated gene editing was barely observed in HSV-1 genomes from any of the treated mice, even when different AAV serotype vectors and several strong promoters were used to improve the transduction efficiency and increase the expression of Cas9 and gRNAs [26,27]. These results indicated that AAV-Cas9 targeting only mediates weak gene editing of HSV-1 in vivo in the mouse model [26].

## 4. Development of Targeted Nucleases against HSV-1 Infection

Homing endonucleases are highly specific nucleases that recognize and cleave large DNA sequences, leading to generation of DNA double strand breaks [13]. In an interesting study, Grosse and colleagues showed that expression of HSV-1-specific HEs could decrease HSV-1 growth in cultured cells [28]. They have previously generated a proprietary collection of ~30,000 engineered endonucleases that were derived from the I-Cre meganuclease [29,30]. Furthermore, they identified more than 500 potential target sites and constructed anti-HSV-1 HEs based on four sites (e.g., *UL19* and *ICP0*).

Using green fluorescence protein (GFP) expression as a marker for cell viability, Grosse and colleagues showed that the constructed anti-HSV-1 HEs exhibited little cytotoxicity [28]. To evaluate the effects of the constructed HEs on blocking HSV-1 infection and replication, cells were first transfected with plasmids containing the expression cassettes for the constructed anti-HSV-1 HEs, and then infected with wild type HSV-1 (SC16 strain) as well as a recombinant HSV-1 containing a *LacZ* reporter. These experiments showed that the constructed anti-HSV HEs were able to inhibit viral replication and growth [28].

Several interesting observations were also reported by Grosse and colleagues. First, the anti-HSV effects (i.e., inhibition of HSV-1 replication and growth) appeared to be specific to the constructed HEs used in the study. This is because no anti-HSV effects were observed in cells expressing control HEs or HEs that targeted non-HSV-1 sequences. Second, the potential mode of action of the constructed anti-HSV-1 HEs was investigated. The authors showed that the anti-HSV-1 meganucleases induced high rates of mutations at their target sites in the HSV-1 genome. When HEs were not present, indel mutations were barely detected. However, mutation frequencies increased when HEs were present. These observations indicated that cleavage by HEs occurred and were consistent with the notion that the mode of the action of these HEs is via cleaving of the viral genomic DNA [28].

Following these interesting observations, Jerome and colleagues investigated the ability of engineered anti-HSV HEs to inhibit HSV-1 latent infections in a unique tissue culture model [31]. In this in vitro tissue culture model, latent HSV-1 infection was established in primary human fibroblasts in the presence of interferon-α (IFN-α) and acyclovir using the GFP-expressing HSV-1 F*ΔUS5* virus [32]. Anti-HSV-1 HEs were constructed to target the region of *UL19*, which encodes viral essential major capsid protein [31]. The 3′-5′ exonuclease Trex2 was also included in the study. Trex2 cleaves 3′ overhangs generated by the HE-induced DNA double strand break, and can improve the ability of HEs to increase the frequency of targeted mutagenesis in cells [33,34]. Self-complementary adeno-associated virus (scAAV) vectors were used to deliver the HE and Trex2 expression cassettes to the cells latently infected with HSV-1 [31].

Human primary fibroblasts exposed to the constructed anti-HSV-1 HEs did not exhibit any cytotoxicity based on two different assays for level of cell death. Exposure to anti-HSV-1 HEs and Trex2 did not result in a detectable degradation of latent HSV-1 genomes [31]. Experiments were also carried out to determine if HE-mediated editing affected HSV-1 reactivation from latent infection. These results suggested that exposure to HEs in viral latently infected cells yielded cleavage of the latent HSV-1 genome and reduced the number of HSV-1 intact DNA genome for reactivation, leading to lack of infectious virions in the supernatant [31].

Using both models of in vitro culture neurons and mice infected with HSV-1, Jerome and colleagues further determined if exposure to HEs had any effect on HSV-1 latent infection in clinically relevant cells and in an animal model in vivo [27]. They initially screened different AAV serotype vectors and promoter expression cassettes for delivery and expression of HEs in cultured trigeminal ganglia (TG) neurons [35,36]. Two I-Cre-derived HEs were constructed to target the sequences of *UL19* and *UL30* [27]. In addition, the 3′-5′ exonuclease Trex2 was also included in the study. To co-deliver the anti-HSV-1 HEs and Trex2, two separate scAAV8 vectors were used. High levels of co-transduction could be achieved in primary trigeminal ganglia (TG) neurons with these AAV8-based vectors [27]. A reduction of 50%–90% in viral production was found in cells expressing the HEs compared to the control cells. Mutagenesis of viral genomes was compared in neuronal cultures from mouse trigeminal ganglia neurons obtained from 7 (acute phase), 14 (late acute/early latent phase), and 32 (latent phase) days after HSV-1 infection. Mutation levels did not vary, suggesting that the HSV-1 DNAs were similarly susceptible to HE-directed editing during all phases of infection [27].

To investigate if HEs had any effects on HSV-1 infection in animals in vivo, anti-HSV-1 HEs were delivered by intradermal whisker pad injection of AAV vectors, which efficiently transduced sensory trigeminal ganglia (TG) neurons in vivo [27]. In these experiments, mice were infected with HSV-1 via ocular inoculation, and then injected with AAV vectors containing HEs and Trex2 expression cassettes. Droplet digital PCR assays confirmed the efficient transduction of AAV vectors and the expression of HEs and Trex2. Clonal sequencing of the regions around the target sites as well as next generation sequencing analyses indicated the presence of deletions of 1 to 14 bp in neurons from mice expressing the HEs. Similar levels of latent viral genomes were observed in all treated or untreated animals, suggesting that the HE editing did not decrease the levels of latent HSV-1 genomes [27].

To study HSV-1 reactivation from HE-treated mice, TGs were collected from HE-treated mice latently infected with HSV-1 and explanted into culture wells containing Vero cell. A significant delay in the increase of viral DNA copy number and infectious virions was found in TGs from the HE-treated mice compared to those from control mice. These results provided the first direct evidence of the use of a homing endonuclease as an antiviral agent to treat an HSV-1 latent infection in vivo [27].

In a more recent study, Jerome and colleagues studied the effects of HE and Cas9/gRNA approaches on elimination of latent HSV in vivo in the mouse model of ocular HSV-1 infection [26]. Mice establishing HSV-1 latent infections were transduced with scAAV8-based vectors carrying the expression cassettes for HEs targeting *UL19* and *UL30*, and *ICP0*. Superior cervical (SCG) and trigeminal ganglia (TG) were collected for analysis. In agreement with their previous results [27], animals receiving a single HE showed modest levels of gene editing of HSV-1 target sites, and neither SCG nor TG showed a detectable reduction in ganglionic HSV-1 load compared to control mice [26]. However, when infected mice were treated with dual-HE therapy, a significant decrease in HSV-1 loads was detected in the SCG and TG [26].

Dual meganuclease treatment was performed by treating mice with two different nucleases, one that targets UL19 and one that targets UL30. To determine the impact of this dual-meganuclease treatment on HSV-1 reactivation from ganglia of treated mice, the collected SCG and TG tissues were subjected to explant reactivation. A significant reduction in HSV-1 genomes in reactivated ganglia was detected in dual-HE-treated mice compared to untreated animals. Furthermore, next generation sequencing analysis revealed higher levels of gene editing and mutations in the reactivated HSV-1 genomes from mice treated with dual-HE compared to those from single HE-treated mice or control mice. A combination of AAV serotype vectors was tested to target all HSV-infected neurons in both autonomic and sensory ganglia and improve transduction efficiency in SCG and TG neurons in vivo. These results showed that dual-HE therapy delivered by a combination of specific types of AAV vectors led to the highest loss of latent viral gnomes from SCG with the greatest decrease in viral load [26].

## 5. Current Limitations/Challenges and Future Considerations

Recent studies on using genome editing-based methods in anti-HSV-1 applications have generated substantial enthusiasm and excitement about applying these approaches to the prevention and treatment of HSV-1 associated diseases (Table 1). Further studies may be needed to address the current limitations/challenges and develop these approaches with the following considerations.

### 5.1. Targeting Efficiency of the Genome Editing Methods

The efficacy of these genome editing methods in editing the HSV-1 genomes during infection is one of the most important factors in practically applying them to the treatment of HSV-1 infections. Extensive research has been carried out to understand the biochemistry and enzymology of HEs [10,13]. Moreover, much progress has recently been made in understanding the mechanism of how Cas9 and a gRNA recognize a DNA target and efficiently generate a cleavage at the target site [9,12]. These results should facilitate the design and construction of HEs and gRNAs with better targeting activity. Furthermore, many new CRISPR/Cas systems have recently been identified from various bacteria and their activities for editing DNA targets have been or are being investigated [9,12]. These studies should lead to the development of new and better HE and Cas9/gRNA systems for anti-HSV-1 applications.

Recent results showed that simultaneous dual or multiplexed HE or Cas9/gRNA targeting on two or more DNA regions yields much better effects than genome editing targeting a single site [15,17,26,27]. Dual and multiplex targeting not only generates multiple cleavages on the DNA targets but also potentially leads to loss and more substantial deletion of the DNA targets. In contrast, a single HE or Cas9/gRNA targeting generates one cleavage, which can be repaired and usually does not lead to a loss or substantial deletion of the target molecules. It would be ideal to choose a gene target that is present more than once in the viral genome (e.g., *ICP0* and *ICP4*).

The unique aspects of HSV-1 infections and pathogenesis should also be taken into consideration when genome editing methods are used. HSV-1 DNA during latency appears to have a more compacted chromatin structure than the replicating viral DNA, leading to less Cas9/gRNA access to DNA target sites [18,19,20]. CRISPR/Cas9 can edit both non-replicating (input or latent) and replicating genomes, although it mutates the replicating genomes much more efficiently [17]. Thus, improving the access of CRISPR/Cas9 to HSV-1 latent genome target sites may increase the editing efficacy and enhance the reduction of HSV-1 latent infection.

HE-mediated targeting did not appear to be significantly influenced by the chromatin structure, as efficient HE-mediated genome editing of HSV-1 was observed in both lytic and latent infections [26,27]. These results suggested that HE and Cas9/gRNA systems exhibited different genome editing efficiency when they were used to target latent and lytic genomes. Furthermore, when they are used to target the same regions of latent HSV-1 genomes in a HSV-1 ocular mouse model, HE-mediated targeting approaches appeared to be much more effective in generating mutations at the target HSV-1 latent DNA and reducing reactivation than the Cas9/gRNA-mediated approaches [26]. These results suggest that the unique aspect of HSV-1 latent infections should be considered when different genome editing methods are used for anti-HSV-1 applications.

### 5.2. Off-Target Effect and Specificity of the Genome Editing Methods

One of the most important concerns about anti-HSV-1 genome editing approaches is their potential toxicity, which could result from long-term expression of the Cas9/gRNAs and HEs in the target tissues and cells. Recent studies suggest that the Cas9/gRNA and HE systems lack adverse effects upon cell viability and processes including the cell cycle, do not induce apoptosis, and do not jeopardize cell viability [14,15,16,17,26,27,28,31].

Three issues should be considered in order to improve the safety of these genome-editing tools and minimize the potential toxicity or off-target side effects. First, sustained expression of these genome editing components could result in off-site cleavage, potentially resulting in mutations or gross chromosomal rearrangements [37]. One way to minimize this effect would be to control the Cas9/gRNA and HE expression and limit it to a critical period of time. Inducible HEs, resulting from fusions with the ligand binding domain of nuclear receptors, have been reported [38,39]. Similarly, the expression of Cas9/gRNA can be modulated through various inducible expression systems.

Second, additional efforts should be made to engineer the Cas9/gRNAs and HEs in order to improve the sequence specificity of these genome editing methods. Extensive studies have been performed to understand the molecular mechanism of how Cas9/gRNAs and HEs interact with their substrate specifically and generate the cleavage [8,9,10,11]. Molecular engineering of these enzymes based on our understanding of their substrate recognition should lead to the generation of novel genome editing enzymes that exhibit high sequence specificity.

Third, serious consideration should be given to the choice of the genes and DNA sequences to be targeted by the genome editing methods. To achieve minimal off-target effects, bioinformatics screening should be vigorously performed using the strictest and most stringent criteria to avoid effects on human translated genomic sites and exclude any transcription DNA motifs. Careful design and choice of the target sites would also make it possible to construct the HE and gRNA easily.

### 5.3. Delivery and Expression of the Genome Editing Components In Vivo

Delivery of the genome editing components and their efficient expression in the delivered tissues and cells are critical for the development of these approaches against HSV-1 infection. There are several virus-based systems available for delivering Cas9/gRNA and HE expression cassettes, including lentivirus, AAV, and modified HSV-1 vectors. Non-viral delivery systems including nanoparticles and extracellular vesicles may also be used. Vectorization to internal structures such as the trigeminal ganglia might be harder than delivery into external tissues or structures commonly associated with HSV-1 infections, such as lips and superficial ocular tissues. Even in easily accessible tissues, the ability of Cas9/gRNA and HE to be effectively used as a stand-alone treatment will need to be evaluated since current vectorization techniques do not allow in vivo vectorization of the large majority of cells. Currently only the AAV systems have been used to deliver genome-editing tools against HSV-1 infections in vivo. Further studies with the other delivery vectors and systems should facilitate the development of better delivery methods for genome-editing approaches against HSV infections.

Different promoter and expression systems have been explored for efficient expression of Cas9/gRNA and HEs in tissues and cells clinically relevant to HSV-1 infection [14,17,26]. The expression of Cas9/gRNAs and HEs can be modulated through various inducible expression systems to control their expression or to expedite Cas9 turnover, which helps to limit off-target effects. Combining the genome-editing strategy with traditional standard anti-HSV-1 therapy such as acyclovir may represent an attractive and promising approach as it may yield potentially synergistic effects that provide more protection than current approaches. Further studies on these issues will facilitate the development of genome editing approaches against HSV-1 infections.

## Figures and Tables

**Table 1:** List of HSV-1 genes targeted by CRISPR/Cas9 or HE strategies and their impact on HSV-1 replication and persistence.

Targeting Method	Delivery Vector	Infection Model	Target Gene(s)	Outcome	Reference
CRISPR/Cas9	Lentivirus	In vitro (TC620 cells)	*ICP0*	Significantly decreased HSV-1 replication	[14]
			*ICP0 + ICP4; ICP0 + ICP27; ICP4 + ICP27*	Drastically reduced HSV-1 replication	
CRISPR/Cas9	Lentivirus	In vitro (Vero cells)	*UL15; UL27; UL29; UL30; UL36; UL37; UL42; UL5; UL52; UL8; UL54; UL9*	Impaired HSV-1 replication	[15]
			*US3; US8*	Impaired HSV-1 replication to a lesser extent	
			*UL29 + UL52; UL8 + UL29*	Completely impaired HSV-1 replication	
CRISPR/Cas9	Plasmid transfection	In vitro (Vero cells)	*UL29 + UL52; UL8 + UL29*	Completely impaired HSV-1 replication	[16]
CRISPR/Cas9	Lentivirus	In vitro (HFF cells)	*UL29; UL30; UL54; ICP4*	Impaired HSV-1 replication Reduced reactivation of quiescent HSV-1 genome	[17]
CRISPR/Cas9	AAV	In vivo (Ocular HSV mouse model)	*UL54 + UL30*	Genome editing too low to observe effects	[26]
HE	AAV	In vivo (Ocular HSV mouse model)	*UL19; UL30*	Decreased HSV-1 load	[26]
			*UL19 + UL30*	Significantly decreased HSV-1 load	
HE	AAV	In vitro (TG neurons)	*UL19; UL30*	Reduced HSV-1 production	[27]
		In vivo (Latent HSV mouse model)		Delayed reactivation of latent HSV-1	
HE	Plasmid transfection	In vitro (COS-7 cells)	*US2; UL19; ICP0*	Inhibited HSV-1 infection and replication	[28]
HE	AAV	In vitro (HF cells)	*UL19*	Decreased HSV-1 replication after reactivation	[31]

## References

[1] Pellett P.E., Roizman B., Knipe D.M., Howley P.M., Cohen J.I., Griffin D.E., Lamb R.A., Martin M.A., Racaniello V.R., Roizman B. (2013). Herpesviridae. Fields Virology.

[2] Roizman B., Knipe D.M., Whitley R.J., Knipe D.M., Howley P.M., Cohen J.I., Griffin D.E., Lamb R.A., Martin M.A., Racaniello V.R., Roizman B. (2013). Herpes simplex viruses. Fields Virology.

[3] Liu F., Zhou Z.H., Arvin A., Campadelli-Fiume G., Mocarski E., Moore P.S., Roizman B., Whitley R., Yamanishi K. (2007). Comparative virion structures of human herpesviruses. Human Herpesviruses: Biology, Therapy, and Immunoprophylaxis.

[4] Kennedy P.G., Steiner I. (2013). Recent issues in herpes simplex encephalitis. J. Neurovirol..

[5] Whitley R.J., Roizman B. (2001). Herpes simplex virus infections. Lancet.

[6] Gupta R., Wald A., Krantz E., Selke S., Warren T., Vargas-Cortes M., Miller G., Corey L. (2004). Valacyclovir and acyclovir for suppression of shedding of herpes simplex virus in the genital tract. J. Infect. Dis..

[7] Mertz G.J., Jones C.C., Mills J., Fife K.H., Lemon S.M., Stapleton J.T., Hill E.L., Davis L.G. (1988). Long-term acyclovir suppression of frequently recurring genital herpes simplex virus infection. A multicenter double-blind trial. JAMA.

[8] Buchholz F., Hauber J. (2016). Antiviral therapy of persistent viral infection using genome editing. Curr. Opin. Virol..

[9] Doudna J.A., Charpentier E. (2014). Genome Editing. The new frontier of genome engineering with CRISPR-Cas9. Science.

[10] Gaj T., Gersbach C.A., Barbas C.F. (2013). ZFN, TALEN, and CRISPR/Cas-based methods for genome engineering. Trends Biotechnol..

[11] Weber N.D., Aubert M., Dang C.H., Stone D., Jerome K.R. (2014). DNA cleavage enzymes for treatment of persistent viral infections: Recent advances and the pathway forward. Virology.

[12] Doudna J.A. (2020). The promise and challenge of therapeutic genome editing. Nature.

[13] Chevalier B.S., Stoddard B.L. (2001). Homing endonucleases: Structural and functional insight into the catalysts of intron/intein mobility. Nucleic Acids Res..

[14] Roehm P.C., Shekarabi M., Wollebo H.S., Bellizzi A., He L., Salkind J., Khalili K. (2016). Inhibition of HSV-1 Replication by Gene Editing Strategy. Sci. Rep..

[15] van Diemen F.R., Kruse E.M., Hooykaas M.J., Bruggeling C.E., Schurch A.C., van Ham P.M., Imhof S.M., Nijhuis M., Wiertz E.J., Lebbink R.J. (2016). CRISPR/Cas9-Mediated Genome Editing of Herpesviruses Limits Productive and Latent Infections. PLoS Pathog..

[16] Karpov D.S., Karpov V.L., Klimova R.R., Demidova N.A., Kushch A.A. (2019). A Plasmid-Expressed CRISPR/Cas9 System Suppresses Replication of HSV Type I in a Vero Cell Culture. Mol. Biol..

[17] Oh H.S., Neuhausser W.M., Eggan P., Angelova M., Kirchner R., Eggan K.C., Knipe D.M. (2019). Herpesviral lytic gene functions render the viral genome susceptible to novel editing by CRISPR/Cas9. eLife.

[18] Chen X., Rinsma M., Janssen J.M., Liu J., Maggio I., Goncalves M.A. (2016). Probing the impact of chromatin conformation on genome editing tools. Nucleic Acids Res..

[19] Horlbeck M.A., Witkowsky L.B., Guglielmi B., Replogle J.M., Gilbert L.A., Villalta J.E., Torigoe S.E., Tjian R., Weissman J.S. (2016). Nucleosomes impede Cas9 access to DNA in vivo and in vitro. eLife.

[20] Hsu P.D., Scott D.A., Weinstein J.A., Ran F.A., Konermann S., Agarwala V., Li Y., Fine E.J., Wu X., Shalem O. (2013). DNA targeting specificity of RNA-guided Cas9 nucleases. Nat. Biotechnol..

[21] Knipe D.M., Cliffe A. (2008). Chromatin control of herpes simplex virus lytic and latent infection. Nat. Rev. Microbiol..

[22] Ran F.A., Cong L., Yan W.X., Scott D.A., Gootenberg J.S., Kriz A.J., Zetsche B., Shalem O., Wu X., Makarova K.S. (2015). In vivo genome editing using Staphylococcus aureus Cas9. Nature.

[23] Ferenczy M.W., DeLuca N.A. (2011). Reversal of heterochromatic silencing of quiescent herpes simplex virus type 1 by ICP0. J. Virol..

[24] Cliffe A.R., Garber D.A., Knipe D.M. (2009). Transcription of the herpes simplex virus latency-associated transcript promotes the formation of facultative heterochromatin on lytic promoters. J. Virol..

[25] Wang Q.Y., Zhou C., Johnson K.E., Colgrove R.C., Coen D.M., Knipe D.M. (2005). Herpesviral latency-associated transcript gene promotes assembly of heterochromatin on viral lytic-gene promoters in latent infection. Proc. Natl. Acad. Sci. USA.

[26] Aubert M., Strongin D.E., Roychoudhury P., Loprieno M.A., Haick A.K., Klouser L.M., Stensland L., Huang M.L., Makhsous N., Tait A. (2020). Gene editing and elimination of latent herpes simplex virus in vivo. Nat. Commun..

[27] Aubert M., Madden E.A., Loprieno M., DeSilva Feelixge H.S., Stensland L., Huang M.L., Greninger A.L., Roychoudhury P., Niyonzima N., Nguyen T. (2016). In vivo disruption of latent HSV by designer endonuclease therapy. JCI Insight.

[28] Grosse S., Huot N., Mahiet C., Arnould S., Barradeau S., Clerre D.L., Chion-Sotinel I., Jacqmarcq C., Chapellier B., Ergani A. (2011). Meganuclease-mediated Inhibition of HSV1 Infection in Cultured Cells. Mol. Ther..

[29] Arnould S., Chames P., Perez C., Lacroix E., Duclert A., Epinat J.C., Stricher F., Petit A.S., Patin A., Guillier S. (2006). Engineering of large numbers of highly specific homing endonucleases that induce recombination on novel DNA targets. J. Mol. Biol..

[30] Smith J., Grizot S., Arnould S., Duclert A., Epinat J.C., Chames P., Prieto J., Redondo P., Blanco F.J., Bravo J. (2006). A combinatorial approach to create artificial homing endonucleases cleaving chosen sequences. Nucleic Acids Res..

[31] Aubert M., Boyle N.M., Stone D., Stensland L., Huang M.L., Magaret A.S., Galetto R., Rawlings D.J., Scharenberg A.M., Jerome K.R. (2014). In vitro Inactivation of Latent HSV by Targeted Mutagenesis Using an HSV-specific Homing Endonuclease. Mol. Ther. Nucleic Acids.

[32] Wigdahl B.L., Scheck A.C., De Clercq E., Rapp F. (1982). High efficiency latency and activation of herpes simplex virus in human cells. Science.

[33] Certo M.T., Gwiazda K.S., Kuhar R., Sather B., Curinga G., Mandt T., Brault M., Lambert A.R., Baxter S.K., Jacoby K. (2012). Coupling endonucleases with DNA end-processing enzymes to drive gene disruption. Nat. Methods.

[34] Delacote F., Perez C., Guyot V., Duhamel M., Rochon C., Ollivier N., Macmaster R., Silva G.H., Paques F., Daboussi F. (2013). High frequency targeted mutagenesis using engineered endonucleases and DNA-end processing enzymes. PLoS ONE.

[35] Jacques S.J., Ahmed Z., Forbes A., Douglas M.R., Vigenswara V., Berry M., Logan A. (2012). AAV8 (gfp) preferentially targets large diameter dorsal root ganglion neurones after both intra-dorsal root ganglion and intrathecal injection. Mol. Cell Neurosci..

[36] Mason M.R., Ehlert E.M., Eggers R., Pool C.W., Hermening S., Huseinovic A., Timmermans E., Blits B., Verhaagen J. (2010). Comparison of AAV serotypes for gene delivery to dorsal root ganglion neurons. Mol. Ther..

[37] Brunet E., Simsek D., Tomishima M., DeKelver R., Choi V.M., Gregory P., Urnov F., Weinstock D.M., Jasin M. (2009). Chromosomal translocations induced at specified loci in human stem cells. Proc. Natl. Acad. Sci. USA.

[38] Bennardo N., Cheng A., Huang N., Stark J.M. (2008). Alternative-NHEJ is a mechanistically distinct pathway of mammalian chromosome break repair. PLoS Genet..

[39] Soutoglou E., Dorn J.F., Sengupta K., Jasin M., Nussenzweig A., Ried T., Danuser G., Misteli T. (2007). Positional stability of single double-strand breaks in mammalian cells. Nat. Cell Biol..

